# Case report: Two unexpected cases of DGUOK-related mitochondrial DNA depletion syndrome presenting with hyperinsulinemic hypoglycemia

**DOI:** 10.3389/fendo.2023.1268135

**Published:** 2023-11-01

**Authors:** Herodes Guzman, Sahr Yazdani, Jennifer L. Harmon, Kimberly A. Chapman, Bernadette Vitola, Louise Pyle, Heather McKnight, Winnie Sigal, Katherine Lord, Diva D. De Leon, Nadia Merchant, Rebecca Ganetzky

**Affiliations:** ^1^Division of Endocrinology and Diabetes, Children’s Hospital of Philadelphia, Philadelphia, PA, United States; ^2^Division of Genetics, Children’s Hospital of Philadelphia, Philadelphia, PA, United States; ^3^Division of General Pediatrics, Children’s Hospital of Philadelphia, Philadelphia, PA, United States; ^4^Rare Disease Institute, Children’s National Hospital, Washington, DC, United States; ^5^Division of Gastroenterology, Hepatology and Nutrition, Children’s National Hospital, Washington, DC, United States; ^6^Transplant Institute, MedStar Georgetown University Hospital, Washington, DC, United States; ^7^Department of Pediatrics, Perelman School of Medicine at the University of Pennsylvania, Philadelphia, PA, United States; ^8^Division of Endocrinology and Diabetes, Children’s National Hospital, Washington, DC, United States

**Keywords:** hypoglycemia, exome, mitochondria, DGUOK, hyperinsulinism

## Abstract

Timely diagnosis of persistent neonatal hypoglycemia is critical to prevent neurological sequelae, but diagnosis is complicated by the heterogenicity of the causes. We discuss two cases at separate institutions in which clinical management was fundamentally altered by the results of molecular genetic testing. In both patients, critical samples demonstrated hypoketotic hypoglycemia and a partial glycemic response to glucagon stimulation, thereby suggesting hyperinsulinism (HI). However, due to rapid genetic testing, both patients were found to have deoxyguanosine kinase (DGUOK)-related mitochondrial DNA depletion syndrome, an unexpected diagnosis. Patients with this disease typically present with either hepatocerebral disease in the neonatal period or isolated hepatic failure in infancy. The characteristic features involved in the hepatocerebral form of the disease include lactic acidosis, hypoglycemia, cholestasis, progressive liver failure, and increasing neurologic dysfunction. Those with isolated liver involvement experience hepatomegaly, cholestasis, and liver failure. Although liver transplantation is considered, research has demonstrated that for patients with DGUOK-related mitochondrial DNA depletion syndrome and neurologic symptoms, early demise occurs. Our report advocates for the prompt initiation of genetic testing in patients presenting with persistent neonatal hypoglycemia and for the incorporation of mitochondrial DNA depletion syndromes in the differential diagnosis of HI.

## 1 Introduction

Timely diagnosis of persistent neonatal hypoglycemia is critical to prevent neurological sequelae, but diagnosis is complicated by the heterogenicity of the causes. Deoxyguanosine kinase (DGUOK)-related mitochondrial DNA depletion syndrome (OMIM# 251880) or DGUOK deficiency can present with hypoglycemia but has additional phenotypic manifestations. This autosomal recessive mitochondrial disorder results from biallelic pathogenic variants in the *DGUOK* nuclear gene, which encodes the deoxyguanosine kinase involved in mitochondrial DNA (mtDNA) maintenance. Deficiency in DGUOK leads to impaired mitochondrial deoxynucleotide triphosphate production, resulting in mtDNA depletion and respiratory chain dysfunction (1, 2).

Patients with DGUOK deficiency typically present in two ways: multi-systemic disease (hepatocerebral type) in the neonatal period or isolated hepatic failure in infancy. In the multi-systemic form, psychomotor delay, rotary nystagmus, and hypotonia can be observed in addition to severe and progressive liver dysfunction. Those with isolated liver involvement suffer from hepatomegaly, cholestasis, and liver failure (2).

Diagnosis is facilitated by molecular genetic testing (3), which is usually done after finding biochemical derangements including conjugated hyperbilirubinemia, elevated gamma-glutamyltransferase, hepatitis with associated tyrosinemia, hypoglycemia, and lactic acidosis. Coagulopathies may also be observed secondary to liver failure. Despite the significance of presentation, head imaging is often normal. If tissue is collected, liver and muscle samples typically show a reduced mtDNA copy number, combined deficiency of liver respiratory chain complexes I, III, and IV, and a greater number of mitochondria with abnormal cristae on liver electron microscopy (2). Most patients with multi-systemic involvement pass away from liver failure at around 1-2 years of life (2, 4, 5).

In this report, we present two cases of persistent hypoketotic hypoglycemia initially undergoing work-up for hyperinsulinism (HI). Rapid genetic testing was critical in facilitating the unexpected diagnosis of DGUOK deficiency in each patient, fundamentally altering their clinical management. As HI has been only rarely reported in DGUOK deficiency (6, 7), our two patients establish a pattern of hyperinsulinemia in mtDNA depletion syndromes, warranting consideration of these disorders in the differential diagnosis of HI.

## 2 Case reports

Case 1: The first patient was a full-term male born at an outside hospital via vacuum-assisted delivery following an uncomplicated pregnancy with APGARS of 8 and 9 at 1 and 5 minutes of life, respectively, and a birth weight of 2,349 grams (1st percentile). This was the parents first child. On day of life (DOL) 0, he experienced hypoglycemia (plasma glucose [PG] 34 mg/dL; reference range: 70-99 mg/dL) and profound lactic acidosis (lactate 16.2 mmol/L; reference range: ≤1.5 mmol/L) with pH 7.29 and bicarbonate 10 mmol/L (reference range: 20-26 mmol/L). On physical examination, he was noted to have penoscrotal hypospadias and significant tachypnea. He was initiated on high-flow nasal cannula, ampicillin, gentamicin, and phototherapy given unconjugated hyperbilirubinemia at birth. He was then transferred to a quaternary care NICU for further management, including treatment of developing coagulopathy.

Further evaluation demonstrated persistently elevated plasma lactate, elevated plasma alanine, proline, and tyrosine, and low serum beta-hydroxybutyrate. Although the newborn screen was positive for an abnormally elevated tyrosine, succinylacetone was not detected in urine organic acids. The infant’s normal acylcarnitine profile was not suggestive of a fatty acid oxidation disorder. A metabolic hypoglycemia genetic panel and trio whole exome sequencing were sent on DOL 4 and DOL 6, respectively. The family history was negative for acute liver failure, hypoglycemia, or sudden infant death. The parents were of Mexican ancestry and nonconsanguineous, though their families are from the same province in Mexico. To further characterize the patient’s hypoglycemia, he underwent a diagnostic fast that demonstrated hypoketotic hypoglycemia and a partial glycemic response to glucagon stimulation, which was suggestive of HI although his persistent lactatemia was inconsistent with this diagnosis.

The infant was transferred to the endocrinology service on DOL 22 for continued hypoglycemia and received dextrose-containing fluids at a glucose infusion rate (GIR) of 8-10.5 mg/kg/min. On DOL 25, his metabolic hypoglycemia genetic panel resulted with a biparentally-inherited homozygous likely pathogenic variant in the *DGUOK* gene (c.749T>C, p.Leu250Ser), consistent with DGUOK deficiency. Notably, whole exome sequencing was not expected to produce a result for 2 more weeks. In light of this diagnosis, mitochondrial medicine, transplant hepatology, and palliative care were consulted. The patient was started on a mitochondrial cocktail of allopurinol and inosine, and liver transplantation was discussed. For his persistent hypoglycemia, a gastrostomy tube was placed for continuous enteral dextrose fluid infusion. With confirmation of this diagnosis and negative workup for congenital adrenal insufficiency, further management of his under-virilized genitalia was deferred. He was discharged home on DOL 49 with close outpatient follow-up.

At home, the family reported intermittent hypoglycemia requiring up-titration of the infant’s enteral dextrose. By 4 months of age, he developed nystagmus, rendering a liver transplant inadmissible. Thereafter, he was admitted multiple times for worsening abdominal distension, ascites, and coagulopathy, eventually succumbing to his disease at 5 months of age.

Case 2: The second patient is a full-term male born at an outside hospital via precipitous spontaneous vaginal delivery to a mother with gestational diabetes with APGARS of 8 and 9 at 1 and 5 minutes of life, respectively, and birth weight of 2,680 grams (5th percentile). To our knowledge, the mother had insulin-dependent gestational diabetes that arose at 34 weeks gestation. Since the family had arrived in the United States at 32 weeks gestation, the mother’s glycemic control was not known. After admission, the patient’s plasma glucose levels progressively fell from 73 mg/dL to 50 mg/dL (reference range: 70-99 mg/dL). At 19 hours of life, he became hypothermic, tachypneic, and increasingly lethargic with multiple poor feeding attempts. He was transferred to the NICU where his plasma glucose returned undetectable. He was subsequently started on dextrose-containing fluids as well as IV ampicillin and gentamicin for early-onset sepsis.

On DOL 2, the patient was noted to have significant lactic acidosis (lactate 20 mmol/L; reference range: ≤1.5 mmol/L) with pH 7.13 and bicarbonate <15 mmol/L (reference range: 20-26 mmol/L). Additionally, he had multiple increased laboratory markers of liver dysfunction. Due to persistent hypoglycemia, GIR in his total parenteral nutrition (TPN) was increased to 5.5 mg/kg/min. On DOL 3, feeding initiation was stopped to limit protein intake given a developing concern for a metabolic disorder with ongoing lactic acidosis and hyperammonemia. Shortly after, he became coagulopathic and was transferred to a quaternary care NICU on DOL 4 for further management.

On DOL 5, metabolic testing in the setting of hypoglycemia revealed persistently elevated lactate, elevated plasma alanine, glycine, proline, and tyrosine, and low serum beta-hydroxybutyrate. Given the patient’s history of a normal newborn metabolic screen and the non-diagnostic pattern of these amino acid elevations in the setting of liver dysfunction, these lab abnormalities were determined to signal liver failure rather than evidence of a metabolic disorder. Feeds, including protein, were slowly reintroduced without a significant increase in ammonia. Due to ongoing lactic acidosis, liver failure with synthetic dysfunction, and persistent hypoglycemia, genetic testing with trio whole exome sequencing was sent. There was no reported family history of acute liver failure, hypoglycemia, or sudden infant death. The parents were of Sudanese ethnicity and fifth cousins. Their only other child is a healthy 8-year-old girl.

Hypoglycemia was noted again with an attempted transition from parenteral to enteral nutrition, so a critical sample was obtained on DOL 18 that revealed hypoketotic hypoglycemia with partial response to glucagon stimulation, suggesting possible HI. This patient was started on diazoxide and chlorothiazide to wean off GIR through the TPN and facilitate the transition to full enteral feeds. However, he demonstrated only a partial response to diazoxide with a continued need for dextrose support.

Whole exome sequencing was reported on DOL 51 with biparentally-inherited homozygous likely pathogenic variants in the *DGUOK* gene (c.757_759del), revealing DGUOK deficiency. Following this diagnosis, diazoxide and chlorothiazide were discontinued. A gastrostomy tube was placed to allow for background continuous formula feeds to prevent hypoglycemia with the addition of PO ad libitum feeds on top. Given the patient’s multisystem disease, hepatology determined him ineligible for liver transplantation.

**Figure 1:**
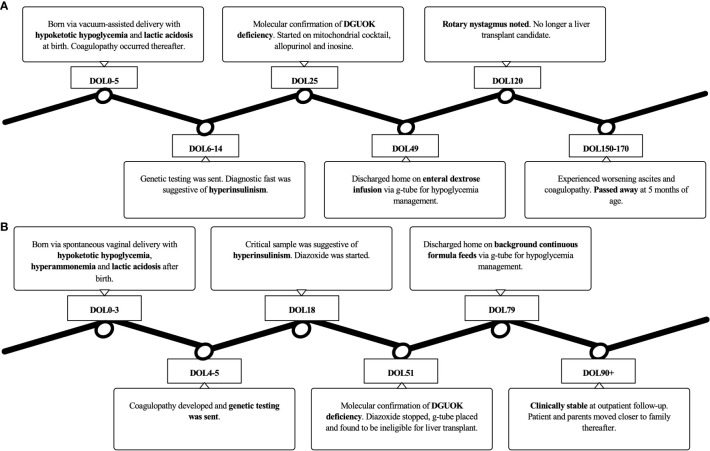
A timeline of the clinical course for **(A)** Patient 1 and **(B)** Patient 2.

After a multidisciplinary discussion, the family decided they wanted to spend time with their infant at home, so palliative care was consulted to facilitate the transition to home care. He was discharged home on DOL 79. At the time of his outpatient follow-up at 3 months of age, he was stable on his discharge regimen with no new symptoms. The family subsequently moved with the patient to be closer to extended family members, and his care was transitioned to local pediatric subspecialists. A timeline of the clinical course for each patient is presented in **Figure 1**.

## 3 Discussion

Rapid genetic testing fundamentally altered the management of both patients diagnosed with a rare mitochondrial disorder. While biochemical studies suggested a diagnosis of congenital HI, molecular genetic testing confirmed mtDNA depletion syndrome from DGUOK deficiency. Of the six other documented patients with the same homozygous variant as our first patient, four of them passed away from liver failure at a couple of months to 2 years of age (8, 9). Notably, one of the patients alive at the time of publication received a liver transplant despite neurologic symptoms and was reported to continue having severe neurologic dysfunction and developmental delay afterward (10). It is unclear whether any of these patients also suffered from hyperinsulinemic hypoglycemia. Similarly, lactic acidosis with rapidly progressive liver failure and neurologic dysfunction were observed in two patients with a heterozygous c.749T>C *DGUOK* missense variant (9, 11). The only other reported heterozygous patient was alive at the time of publication with isolated liver disease (11). Based on this prior variant evidence, phenotypic overlap in our patient, functional data showing significantly reduced enzyme activity and the lack of homozygotes present in the Genome Aggregation Database (gnomAD) with a minor allele frequency of 0.0032% (8/251,466 alleles); this variant was classified as likely pathogenic (8).

In contrast, the c.757_759del variant identified in our second patient has only been reported in one other individual in ClinVar as a variant of uncertain significance. No citations or clinical data are provided for this case. Moreover, functional data on this variant are not available in the literature. No homozygotes are present in gnomAD, and the variant has a minor allele frequency of 0.00040% (1/251,456 alleles). Although specific clinical data were not available from other patients with this variant, this patient’s clinical presentation and disease course were consistent with other cases of DGUOK deficiency.

In both cases, liver transplantation was considered to treat their fulminant liver failure. As of 2020, a total of 20 patients with DGUOK deficiency have undergone liver transplantation and half have died from transplant-related complications (12). Those without neurologic features prior to transplant may still go on to develop progressive neurologic dysfunction, complicating the decision to transplant in any case of DGUOK deficiency (2, 11, 13, 14). In the first patient, his neurologic symptoms precluded this option. Due to a multisystem disease in the second patient, he was also determined to be a poor liver transplant candidate.

Various disorders lead to altered glucose homeostasis, including HI, panhypopituitarism, glycogen storage disorders, fatty acid oxidation defects, ketogenesis or ketolysis defects, disorders of gluconeogenesis, galactosemia, hereditary fructose intolerance, congenital disorders of glycosylation, mitochondrial disease, syndromic hypoglycemia, and select aminoacidopathies (15). Diagnosis is often delayed by the overlapping features seen in these disorders. Biochemical analysis is necessary for phenotyping and stratifying these patients (i.e., ketotic versus non-ketotic hypoglycemia), while molecular genetic testing is often required to confirm a diagnosis (15). Our presented equivocal cases help illustrate situations in which prompt initiation of genetic testing is essential to establishing an effective management plan.

For both of our patients, the diagnosis of HI was considered based on increased glucose requirements, the pattern of hypoketotic hypoglycemia, and the partial glycemic response to glucagon even though the elevated lactate was inconsistent with this disorder. HI is the most common cause of persistent hypoglycemia. It can be secondary to perinatal factors such as maternal diabetes or perinatal stress, or it can be genetic. Several genes are implicated in the development of HI, most commonly pathogenic variants in *ABCC8* and *KCNJ11* (15). Depending on the affected gene and inheritance pattern, responsiveness to medical treatment can vary with some cases requiring pancreatectomy (15, 16). Given these treatment considerations, genetic testing is considered the standard of care in suspected persistent HI. A two-tiered approach expedites molecular diagnosis: diazoxide-unresponsive patients who are more likely to have focal disease undergo Tier 1 testing that investigates for *ABCC8*, *KCNJ11*, and *GCK* mutations with a turnaround time of 5–7 days while diazoxide-responsive patients undergo more comprehensive genetic testing that results in ~4 weeks (16, 17). As nearly 50% of these patients with HI have non-diagnostic genetic testing results, expedited comprehensive genetic testing such as whole exome or genome sequencing may be warranted. This is especially critical in inconclusive cases like our own, in which the genetic diagnosis of DGUOK deficiency drastically altered clinical management and family decision making.

The characteristic features observed in multi-systemic DGUOK deficiency include lactic acidosis, hypoglycemia, cholestasis, progressive liver failure, and increasing neurologic dysfunction (2). However, diagnosis is complicated by phenotypic variability. Depending on the primary metabolic derangements observed, patients with DGUOK deficiency have been misclassified as neonatal hemochromatosis (hyperferritinemia and elevated alpha-fetoprotein) and tyrosinemia type 1 among other disorders (18). Most notably, an emerging feature of DGUOK deficiency is hyperinsulinemic hypoglycemia. In two deceased infants found to have DGUOK deficiency, autopsy tissue specimens revealed pancreatic islet cell hyperplasia suggestive of HI as the cause of their hypoketotic hypoglycemia. When alive, both autopsied patients had inappropriately high insulin levels and lower free fatty acids in the setting of hypoglycemia with a positive glycemic response to glucagon stimulation (6). A recent report of a third infant with DGUOK deficiency and hyperinsulinemic hypoglycemia reveals partial responsiveness to diazoxide therapy at 8 mg/kg/day dosing, which is similar to our second case (7). Our two cases now support a pattern of hyperinsulinemic hypoglycemia in DGUOK deficiency, thus warranting consideration of this mitochondrial disease in the workup of HI. **Table 1** provides a summary of all reported cases of DGUOK deficiency with hyperinsulinemic hypoglycemia to date. It is important to note that we cannot rule out the possibility of perinatal stress-induced HI in the pathophysiology of hypoglycemia. Regardless, if there is evidence of HI, treatment with diazoxide may be of benefit because recurrent hypoketotic hypoglycemia may be contributing to the progressive neurologic dysfunction observed in these patients. Diazoxide-responsiveness has already been demonstrated in patients with tyrosinemia type 1 and HI, further supporting this treatment strategy for DGUOK deficiency (19).

**Table 1:** All reported cases of deoxyguanosine kinase (DGUOK) deficiency with hyperinsulinemic hypoglycemia to date.

Reference	*DGUOK* variant	Age of presentation	Sex (M/F)	Clinical course	Critical sample^+^	Management
Patient 1	c.749T>C, p.Leu250Ser (homozygous)	At birth	M	Hypoketotic hypoglycemia with lactic acidosis and progressive liver failure resulting in death at 5 months	PG 37 mg/dL, BOHB <0.3 mmol/L, FFA 0.77 mmol/L, insulin 13.7 μIU/mL, c-peptide <0.1 ng/mL, lactate 4.2 mmol/L, delta glucose +22 in response to glucagon	Enteral dextrose infusion
Patient 2	c.757_759del (homozygous)	At birth	M	Hypoketotic hypoglycemia with lactic acidosis and transient hyperammonemia. Progressive liver failure, currently living	PG 38 mg/dL, BOHB <0.1 mmol/L, insulin 13.1 μIU/mL, delta glucose +13 in response to glucagon	Diazoxide, continuous formula feeds
Pronicka **et al. (** 6)	c.766_767insGATT/c.?, p.Phe256X/p.? (heterozygous)	At birth	F	Hypoketotic hypoglycemia with lactic acidosis and progressive liver failure resulting in death at 18 months	PG 22.8 mg/dL, BOHB 0.21 mmol/L, FFA 0.72 mmol/L, insulin 4.3 μIU/mL, delta glucose +77 in response to glucagon	Enteral dextrose infusion
	c.3G>A/c.813_814insTTT, p.Met1Ile/(compound heterozygous)	At birth	F	Hypoketotic hypoglycemia with lactic acidosis and progressive liver failure resulting in death at 6.5 months	PG 21.4 mg/dL, BOHB 0.15 mmol/L, FFA 0.1 mmol/L, insulin 5.7 μIU/mL, delta glucose +108 in response to glucagon	Enteral dextrose infusion
Arya **et al. (** 7)	c.763_766dupGATT, p.Phe256* (homozygous)	At birth	M	Hypoketotic hypoglycemia with lactic acidosis and progressive liver failure resulting in death at 8 months	PG 36 mg/dL, BOHB <0.01 mmol/L, insulin 21.9 μIU/mL, lactate 4.1 mmol/L	Diazoxide, continuous formula feeds

*M, male; F, female; PG, plasma glucose; BOHB, beta-hydroxybutyrate; FFA, non-esterified free fatty acids.

^+^Referenc ranges: PG (70-99 mg/dL), BOHB (≥2.0-5.0 mmol/L), FFA (≥1.5 mmol/L), insulin (≤2 µIU/mL), c-peptide (0.0-3.3 ng/mL), lactate (≤1.5 mmol/L).

Advances in next-generation sequencing have facilitated similar diagnoses in recent years, particularly in critically ill infants and children (20, 21). For example, among 354 infants receiving either early (15 days after study enrollment) or delayed (60 days after enrollment) whole genome sequencing results, twice as many participants with early genetic diagnosis had a change in clinical management, including subspecialty referrals, condition-specific medications, and surgical interventions (22). The overall diagnostic yield of genetic screening in this patient population is 37% with management changes reported in 20-100% of diagnosed patients (23). Not only does genetic testing affect clinical outcomes, but recent analyses also highlight the economic benefits of timely genetic diagnosis (20, 21, 24). By reducing the length of hospital stays, genetic testing can save up to two million US dollars per 100 patients tested (20). Moreover, one study demonstrated that both the public and affected families have a vested interest in rapid genetic testing for critically ill children despite the cost (25).

In conclusion, our experiences reinforce the importance of rapid genetic testing for patients with persistent neonatal hypoglycemia, particularly when biochemical testing is inconclusive. With these two new cases, there are now five patients with DGUOK deficiency and hyperinsulinemic hypoglycemia, extending the differential diagnosis of HI to include mtDNA depletion syndromes and giving further credence to the utility of expedited comprehensive genetic testing in these scenarios. Most significantly, timely molecular confirmation of DGUOK deficiency can shorten the diagnostic odyssey for these patients and allow for the cessation of futile interventions, thus maximizing the time a family has with their affected child.

## 4 Patient perspective

Not provided by the parents of Patient 1. We were unable to reach the parents of Patient 2.

## Data availability statement

The original contributions presented in the study are included in the article/**Supplementary Material**. Further inquiries can be directed to the corresponding author.

## Ethics statement

Written informed consent was obtained from the minors’ legal guardian/next of kin for the publication of this case report.

## Author contributions

HG: Writing – original draft, Writing – review & editing. SY: Writing – original draft, Writing – review & editing. JH: Writing – original draft, Writing – review & editing. KC: Writing – review & editing. BV: Writing – review & editing. LP: Writing – review & editing. HM: Writing – review & editing. WS: Writing – review & editing. KL: Writing – review & editing. DL: Writing – review & editing. NM: Writing – original draft, Writing – review & editing. RG: Writing – review & editing.
