# Supplementary material for: Case report: Two unexpected cases of DGUOK-related mitochondrial DNA depletion syndrome presenting with hyperinsulinemic hypoglycemia
Source: Front Endocrinol (Lausanne). 2023 Nov 1;14:1268135. doi: 10.3389/fendo.2023.1268135 (PMC10646319; doi:10.3389/fendo.2023.1268135)
Supplement: Supplementary file 1 [file DataSheet_1.pdf]

CARE case report guidelines

CARE Checklist of information to include when writing a case report

(cc) BY-NC-ND

Topic	Item	Checklist item description	Reported on Line
Title	1	The diagnosis or intervention of primary focus followed by the words "case report"	*Yes, title line*
Key Words	2	2 to 5 key words that identify diagnoses or interventions in this case report, including "case report"	*Line 23*
Abstract (no references)	3a	Introduction: What is unique about this case and what does it add to the scientific literature?	*Lines 26-31*
	3b	Main symptoms and/or important clinical findings	*Lines 28-29*
	3c	The main diagnoses, therapeutic interventions, and outcomes	*Lines 30-38*
	3d	Conclusion—What is the main "take-away" lesson(s) from this case?	*Lines 38-40*
Introduction	4	One or two paragraphs summarizing why this case is unique (**may include references**)	*Lines 63-68*
Patient Information	5a	De-identified patient specific information.	*Lines 70-74 + 105-109*
	5b	Primary concerns and symptoms of the patient.	*Lines 70-74 + 105-109*
	5c	Medical, family, and psycho-social history including relevant genetic information	*Lines 70 + 105*
	5d	Relevant past interventions with outcomes	*N/A*
Clinical Findings	6	Describe significant physical examination (PE) and important clinical findings.	*Lines 73-74 + 107-108*
Timeline	7	Historical and current information from this episode of care organized as a timeline	*Line 335*
Diagnostic Assessment	8a	Diagnostic testing (such as PE, laboratory testing, imaging, surveys).	*Lines 79-98 + 109-141*
	8b	Diagnostic challenges (such as access to testing, financial, or cultural)	*N/A*
	8c	Diagnosis (including other diagnoses considered)	*Lines 89-91 + 134-135*
	8d	Prognosis (such as staging in oncology) where applicable	*N/A*
Therapeutic Intervention	9a	Types of therapeutic intervention (such as pharmacologic, surgical, preventive, self-care)	*Lines 88-101 + 109-141*
	9b	Administration of therapeutic intervention (such as dosage, strength, duration)	*Lines 89,94-95 + 109-141*
	9c	Changes in therapeutic intervention (with rationale)	*Lines 88-98 + 138-141*
Follow-up and Outcomes	10a	Clinician and patient-assessed outcomes (if available)	*Lines 100-103 + 143-147*
	10b	Important follow-up diagnostic and other test results	*N/A*
	10c	Intervention adherence and tolerability (How was this assessed?)	*N/A*
	10d	Adverse and unanticipated events	*Lines 100-103*
Discussion	11a	A scientific discussion of the strengths AND limitations associated with this case report	*N/A*
	11b	Discussion of the relevant medical literature **with references**.	*Lines 149-226*
	11c	The scientific rationale for any conclusions (including assessment of possible causes)	*Lines 149-234*
	11d	The primary "take-away" lessons of this case report (without references) in a one paragraph conclusion	*Lines 227-234*
Patient Perspective	12	The patient should share their perspective in one to two paragraphs on the treatment(s) they received	*N/A*
Informed Consent	13	Did the patient give informed consent? Please provide if requested	Yes ☒ No ☒

**Explanation in manuscript.*
